# Coaching in undergraduate medical education: a national survey

**DOI:** 10.1080/10872981.2019.1699765

**Published:** 2019-12-03

**Authors:** Margaret Wolff, Maya Hammoud, Sally Santen, Nicole Deiorio, Megan Fix

**Affiliations:** a Emergency Medicine and Pediatrics, University of Michigan Medical School, Ann Arbor, MI, USA; b Obstetrics and Gynecology and Learning Health Sciences, University of Michigan Medical School, Ann Arbor, MI, USA; c Emergency Medicine and Senior Associate Dean of Evaluation, Assessment and Scholarship, Virginia Commonwealth University, Richmond, VA, USA; d Emergency Medicine and Associate Dean of Student Affairs, Virginia Commonwealth University, Richmond, VA, USA; e Surgery, University of Utah School of Medicine, Salt Lake City, UT, USA

**Keywords:** Coaching, advising, mentorship, undergraduate medical education, wellbeing

## Abstract

**Background**: Evidence to support coaching in medical education has grown over the past decade leading educators to search for resources to aid the development of their own coaching programs. A sample of medical schools in the USA were surveyed to describe coaching programs to assist other institutions in the development and implementation of programs.

**Methods**: Participants representing 32 medical schools attending a coaching conference in October 2018 were surveyed via email regarding their undergraduate medical education (UME) coaching programs. The 19-item instrument contained questions on program demographics, program characteristics, coach characteristics, coach training and coach assessment and program evaluation.

**Results**: The response rate was 100% (32/32 programs). Nearly all respondents had a coaching program (53%, 17/32) or were developing one (44%, 14/32) with the majority being implemented in the past five years (82%, 14/17). Professional identity formation (80%, 20/25), professionalism (76%, 19/25), and academic performance (76%, 19/25) were the most commonly identified programmatic goals. The majority of coaches (64%, 16/25) received between 5-25% full time equivalent effort to support their role. Coaches did not formally assess students in any domain at most programs (84%, 21/25) or directly observe their students clinically (76%, 19/25). The majority of programs had formal coach training (88%, 22/25).

**Conclusion**: These results demonstrate that coaching is being used to improve performance, professionalism, and professional identity formation in UME. This sample of coaching programs informs the discussion of coaching in medical education as educators strive to implement effective coaching programs.

## Introduction

Coaching is ubiquitous in high-performance professions such as music and sports and has only gained attention in medical education over the past decade [1,2]. Given the nascent status of coaching in medical education, a universal definition is lacking and the term coaching is often used interchangeably with teaching, advising, and feedback [1,2]. Examples in the medical education literature describe coaching for technical skills, nontechnical skills, wellbeing, and academic performance [1,3]. Best practices have been described, but educators continue to search for resources and real life examples to navigate the development and implementation of coaching programs [3–6]. To address this gap, we sought to describe coaching programs from a convenience sample of medical schools across the USA.

## Methods

### Study design and population

A representative from each of the 32 medical schools participating in American Medical Association’s (AMA) Accelerating Change in Medical Education Consortium attended a coaching thematic conference in October 2018 in Boston, MA. These schools were previously chosen by the AMA to receive innovation grants to redesign curricula for flexible, individualized learning pathways and are diverse in program size, location, and mission (See Table 1) [7]. Following the meeting, participants were surveyed regarding their medical schools’ coaching programs via email between March 2019 and April 2019 using Qualtrics survey software (Qualtrics, Provo, UT). Nonresponders received two email reminders. This survey study was determined to be exempt by the institutional review board at the first author’s institution given no personally identifiable information. Consent was implied by participation.

**Table 1.** School characteristics

	N = 32(%)
Region of USA	
Northeast	8(25)
South	9 (28)
Midwest	9 (28)
West	6 (19)
Context	
Urban	14 (44)
Suburban	12 (38)
Rural	6 (19)
School Size	
≤100/class	4 (13)
>100 to ≤ 150/class	19 (59)
>150/class	9 (28)

## Survey content and administration

The survey instrument was developed by the AMA coaching research task force, and included thought leaders in the field of undergraduate medical education (UME) coaching, education research specialists, and executive coaching. The objective of the survey was deﬁned as describing existing coaching programs in UME to assist other institutions in the development of programs. A literature review was performed and four experts on coaching and medical education (2 affiliated with consortium, 2 not affiliated) were queried to identify coaching concepts and best practices. Next, ﬁve faculty coaches from consortium affiliated schools were asked to define academic coaching in medical education and describe the components of their programs. These factors were then formulated into the survey instrument with the aid of an expert in survey methodology. We sought to ensure content validity with the above process and process response validity by piloting the instrument with the above experts and two faculty involved in graduate medical education coaching, for both content and clarity. A few items were clariﬁed and a concept was added (coach selection) based on feedback. Four of the original pre-testers tested the instrument after incorporating changes. The 19-item instrument contained questions on program demographics and questions focused on the following four domains: program characteristics, coach characteristics, coach training and coach assessment and program evaluation. In addition, respondents were asked to define the dynamics in the coaching relationship using the definitions of coaching (the coach helps the student find a strategy through asking clarifying questions), mentoring (the coach provides information directly based on their own experiences and expertise), and advising (the coach serves as an expert and advises the student on a specific area) [1,3]. These definitions were given without providing the corresponding term.

## Results

The response rate was 100% (32/32 programs). Table 1 details school demographics.

### Coaching program characteristics

Of the surveyed schools, 69% (22/32) identified the description of coaching (the coach helps the student find a strategy through asking clarifying questions) to describe their program. Nearly all respondents had a coaching program (53%, 17/32) or were developing one (44%, 14/32). The majority of established programs were implemented in the past five years (82%, 14/17). The remainder of the analysis focuses on the established programs (17) and those implementing in the next year (8). We excluded schools in early planning stages and one institution not currently planning a program.

Although the respondents reported diverse goals (Table 2), professional identity formation (80%, 20/25), professionalism (76%, 19/25), and academic performance (76%, 19/25) were the most commonly identified programmatic goals. All of the programs had several goals as opposed to a singular focus. Some schools provided suggested topics to cover for each session (52%, 13/25) while others emphasized addressing learning goals each session (28%, 7/25), and other programs left the session content entirely up to the student-coach dyad (20%, 5/25). The content covered is listed in Table 3; respondents selected all that applied to their program. Most coaching programs were mandatory (80%, 20/25) and begin in the preclerkship phase (88%, 22/25).

**Table 2.** Goals of coaching programs

Goal	N = 25 (%)
Professional identity formation	20 (80%)
Academic performance	19 (76%)
Professionalism	19 (76%)
Wellbeing	17 (68%)
Community building	12 (48%)
Leadership	10 (40%)
Development of lifelong learning skills	4 (16%)
Remediation	3 (12%)
Clinical skill development	2 (8%)

**Table 3.** Content of coaching programs

Domain Covered	N = 25(%)
Academic performance	23 (92)
Professional development	22 (88)
Goal setting	22 (88)
Well-being	19 (76)
Reflection	18 (72)
Interpersonal communication	18 (72)
Time management	17 (68)
Clinical performance	15(60)
Specialty selection	10 (40)
Coaching is completely leaner-driven (variable content)	8 (32)
Decision making abilities	1(4)

### Coach characteristics

While the structure of coaching programs varied in terms of number of students assigned to each coach, the majority of coaches (64%, 16/25) received between 5-25% full time equivalent effort to support their role. Coaches were attending physicians (80%, 20/25), residents or fellows (20%, 5/25), and/or non-physicians (40%, 10/25); many programs utilized more than one group for this role. Respondents reported that coaches did not formally assess students in any domain at most programs (84%, 21/25) or directly observe their students clinically (76%, 19/25); see Table 4.

**Table 4.** Coaching program characteristics

	N = 25(%)	Additional information
Mandatory student participation (yes)	20 (80)	
When does coaching start?		
Pre-clerkship	22 (88)	
Clerkship	3(14)	
Post-clerkship	1 (4)	
Who are the coaches?		*Non-physicians included:
Attending physicians	20 (80)	Learning specialists, social workers, basic science faculty, psychologists, senior students
Non-physicians*	10 (40)	
Residents or fellows	2 (8)	
How are coaches assigned?		**Some programs employed purposeful mismatch for pairings while others tried to match based on specialty interest, background, or other interests
Program randomly assigns	12 (41)	
Program intentionally assigns based on student preferences of coach characteristics	4 (16)	
Program intentionally assigns based on program priorities**	4 (16)	
Students select their coach	1 (4)	
FTE Support		***FTE support ranged from 0.05 to 0.25 depending on the number of coachees and the frequency of meetings
Yes***	16 (64)	
No, but they receive faculty development funds No, they do not receive any compensation	4 (16) 5 (25)	

### Coach training, assessment and program evaluation

The majority of programs have formal coach training (88%, 22/25). This ranges from a single session prior to their first coaching session (24%, 6/25) to longitudinal sessions that meet monthly (25%, 5/25). Coaches were evaluated by their learners in 56% (14/25) of the programs with most of these evaluations (86%, 12/14) focusing exclusively on learners’ reactions. The same programs (56%, 14/25) reported evaluating their coaching programs.

## Discussion

Our results demonstrate a shared goal of surveyed schools to improve performance, professionalism, and professional identity formation through coaching programs. We noted variability in regard to the program characteristics, coach characteristics, coach training and assessment and program evaluation. These results do not suggest one particular approach to coaching in undergraduate medical education but rather highlight variables each school can carefully consider when developing a coaching program. This sample of coaching programs can inform the curricular design process and discussion of coaching in medical education as educators strive to implement coaching programs.

Similar to when considering other curricular innovations, educators can follow Kern’s Six-Step Approach for curriculum development to determine if there is a need for a coaching program at their school and if so how best to implement a program [8]. This process should begin with problem identification and a needs assessment to determine if there are unmet student needs that may be filled by a coaching program. The varied areas of focus described by surveyed coaching programs can inform the needs assessment by providing potential areas to explore such as professional identity formation and professionalism. After the local needs are determined, specific goals and objectives can be created and objectives can be matched to specific educational strategies including coaching. The next step of implementation is crucial to the success of the coaching program. This step involves identification of resources (who are the personnel? What time do they have? What is the cost?), obtaining support, and administration of the curriculum, anticipating barriers, and introduction of the curriculum. Finally, the last step is evaluation and feedback. Below we describe several study findings and the potential considerations of each on coaching curriculum development.

Schools had varied goals for their coaching programs, reflecting the local needs of each school. Two of the most common programmatic goals were professional identity formation and professionalism. These skills are traditionally taught in part through role-modeling, making coaching a natural fit [9]. Notably, all of the programs surveyed had multifaceted goals rather than a singular focus such as professionalism or wellbeing. Although this approach makes intuitive sense, literature to date has largely focused on coaching interventions with a singular goal [1]. This finding has important implications for how institutions structure new coaching programs and select their coaches. For example, if a coaching program has multiple goals, coach-coachee dyads will need adequate time to address multiple goals and coaches will need to be well versed in multiple content areas.

After coaching program goals and specific objectives are determined, implementation must be determined. Specifically, who will serve as coaches and what compensation will they receive. The cornerstone of coaching is the coach-coachee relationship. The structure of this relationship is distinctly different from mentoring and advising but definitions may be blurred (Table 5) [1,10]. In this study, the majority of respondents correctly identified the coaching relationship as one in which ‘the coach helps the student find a strategy through asking clarifying questions.’ Coaching program directors should be mindful of the key difference between these roles and include faculty development to address discrepancies. Another important consideration is the timing and the process by which coach-coachee pairings are made. In our sample, most pairings were random and established early in medical school, with the majority occurring prior to the clerkship phase. Programmatic goals, resources, faculty bandwidth, and curricular time likely influence the decision of when to initiate the coaching program.

**Table 5.** Relationships in medical education

	Advising	Mentoring	Sponsorship	Coaching		
				Academic Coaching	Clinical Coaching	Professional Coaching
Focus	Specific event	Career development	Career development	Review of academic performance (objective feedback, evaluations, boards, etc)	Clinical task/skill	Wellness, work/life balance
Control	Advisor	Mentor directed	Sponsor directed	Coachee directed	Coachee directed	Coachee directed
Timeline	Single session	Long term	Long term	Time limited	Time limited	Time limited
Strategy	Give advice	Mentor gives information based on his/her own experience	Sponsor actively advocates for mentee	Help learner by asking clarifying questions	Help learner by asking clarifying questions	Help learner by asking clarifying questions

Another consideration in implementation is coach training. Faculty development was integral to the development of programs in our sample, with most having at least one large session and some with multiple longitudinal sessions. Given that most programs are mandatory for students it is important that coach training be robust to ensure all students experience similar benefits from coaching. Additionally, recent literature suggests that intentionally developing a community of practice for coaches is also desirable [11]. Faculty support ranged from zero to 25% FTE in our sample. Interestingly 18.5% of our coaching sample did not receive any form of compensation, suggesting that coaching has some inherent benefits to faculty, such as connection with students. However, for coaching to continue in medical education, funding for coaches should be secured in some fashion.

The final piece of this iterative process is program evaluation. With the increasing emphasis on coaching in medical education, formal evaluation of coaches and programmatic evaluation is paramount. Although the majority of programs in our sample do evaluate their coaches formally, these tools vary considerably. Many respondents report using brief, locally-developed surveys to determine the feasibility of the program and the reactions to the program. Carney and colleagues developed and validated two surveys (one for coach and one for learner) designed to measure the coaching relationship, processes, and outcomes [10]. Programs can utilize these instruments as a robust starting point.

## Limitations

There are several limitations to our study. Specifically, this convenience sample represents schools in the AMA Change Medical Education consortium, which is biased toward early adoption of innovations including coaching. Our sample does represent wide geographical diversity across the USA, however these are mainly schools that are large academic centers which may not be representative of all medical schools desiring coaching programs. Additionally, there is variability in how coaching is defined.

## Conclusion

Our findings from a geographically diverse US sample of medical schools suggests that coaching is increasing in medical education. The findings of coaching programs’ structure, focus, assessment, faculty development, and budget may help inform the development of other coaching programs.
